# Influence of maintaining apical patency in post-endodontic pain

**DOI:** 10.1186/s12903-021-01632-x

**Published:** 2021-06-02

**Authors:** Snigdha Shubham, Manisha Nepal, Ravish Mishra, Kishor Dutta

**Affiliations:** 1 Department of Conservative Dentistry and Endodontics, Universal College of Medical Sciences, Ranigaon, Bhairahawa, Nepal; 2 Department of Oral and Maxillofacial Surgery, Universal College of Medical Sciences, Ranigaon, Bhairahawa, Nepal; 3 Department of Orthodontics and Dentofacial Orthopaedics, Universal College of Medical Sciences, Ranigaon, Bhairahawa, Nepal

**Keywords:** Apical patency, Multiple visit, Post-operative pain, Single visit

## Abstract

**Background:**

The concept of instrumentation beyond the apical foramen by small flexible file to prevent apical blockage is apical patency. However, this procedure might endow postoperative pain, thus to maintain apical patency or not is the matter of dilemma. Hence, the primary objective of this study was to compare postoperative pain between apical patency and non-patency groups and secondary objective was to evaluate the influence of number of visits, vitality of teeth, group of teeth and preoperative pain on post-operative pain.

**Methods:**

Preselected (n = 178) patients based on group of teeth and status of pulp were randomly divided into 2 groups, apical patency and non-patency which was further treated in either single or multiple visits. After exclusion, 160 patients were included. Each group (n = 80) was subdivided in single visit (n = 40) and multiple visits (n = 40), including vital (n = 20) and non-vital teeth (n = 20) and single-rooted (n = 10) and multiple-rooted teeth (n = 10). Apical patency was maintained with a size 10 K-file during conventional hand filing step-back shaping procedure. Intensity of pain was recorded before treatment and on days 1, 2, and 7 after treatment using Numerical Rating Scale (NRS-11). Statistical analysis was done using Mann–Whitney U test, Spearman correlation and Multiple linear regression analysis.

**Results:**

The primary outcome of this study showed statistically significant difference (*p* < 0.05) in postoperative pain scores between patency and non-patency groups with higher pain scores in patency group on 1st, 2nd and 7th day follow up. The secondary outcome showed postoperative pain in patency-maintained group was influenced by status of the pulp and preoperative pain only. Vital teeth of patency-maintained group treated in multiple visits showed statistically significant (*p* = 0.02) post-operative pain in day 1 follow up. Pre-operative pain showed positive correlation with postoperative pain with statistically significant difference.

**Conclusions:**

Our study concluded that maintenance of apical patency increased postoperative pain. Evaluation of influence of number of visits, status of pulp, group of tooth and preoperative pain revealed status of pulp and preoperative pain as influencing factors for postoperative pain in patency group.

## Background

Root canal treatment is always feared for tiresome procedure and postoperative pain. This has provoked search for the factors increasing the ease of process and decreasing the postoperative pain [1, 2]. Either in disinfection method or maintaining the actual length of canal space there are lot of varied opinion about the protocols to follow. Out of these, maintaining apical patency is also one of the controversies [3].

During the shaping of root canals, pulpal and dentinal debris get collected in the apical third area leading to blockage and loss of working length, transportation, ledge and perforation. Hence, to resolve these issues, Buchanan [4] has proposed a concept of apical patency in which small flexible file is repeatedly extended beyond the apical foramen leaving the foramen patent. Apical Patency according to the Glossary of Endodontic Terms published by the American Association of Endodontists, is defined as a preparation technique in which the apical region of the root canal is maintained as free of debris by recapitulating through the apical constriction with a fine file [5]. To maintain apical patency, size 10 K file is intentionally extended 1mm beyond the working length passively after each instrumentation [4, 6]. To prevent the apical binding and enlarging the apical foramen, size 10 K-file has been used most frequently [7]. Irrigation should be done after the patency file as it will loosen the tissue debris [6].

Apical constriction which is present 0.5 to 1.5 mm above major foramen, is regarded as the reference point for termination of shaping, cleaning and obturation [8]. Predominance of anaerobic microorganisms in apical third, including the cemental canal [9, 10] has led to the idea that the endodontic treatment should not be limited 1 mm short of root apex rather be extended to the full length of canal involving cemental canal i.e. beyond the apical constriction [11, 12]. One of the arguments against this procedure is that a file binding to the foramen which acts like an embolus, increasing the possibility of debris extrusion beyond the apex. Another argument is the severe periapical tissue reaction increasing the chance of postoperative pain and flare-up [13]. Hence, patency concept is controversial and the procedure is taught only in 50 % of U. S. dental schools [7].

There are only few published researches evaluating postoperative pain after maintenance and non-maintenance of apical patency [14–20]. Some of these literatures showed superiority of apical patency group [15, 18], however some studies [14, 16, 17, 19, 20] showed no difference. Hence, the study hypothesizes that there is no difference in post-operative pain between apical patency and non-apical patency group. This study tests equivalence of both groups. Thus, the primary objective of this prospective study was to assess post-operative pain in apical patency and non-patency groups and secondary objective was to assess influence of number of visits, status of pulp, group of teeth and preoperative pain.

## Methods

This research was conducted with the approval of the Institutional Review Committee (IRC number 077/19) in the duration from April 2019 to December 2019.

Endodontic treatment was performed in single or multiple visits of either vital or non-vital tooth and anterior or posterior tooth. Each patient was explained about the aims and design of the study, and informed written consent were obtained before their inclusion. The exclusion criteria were: complex cases such as pulp canal obliteration, procedural accidents, variable anatomy where maintaining patency is difficult, retreatment cases, teeth with periapical radiolucency and swelling, pregnancy, patients who are medically compromised and patients under analgesic medication within last 3 days.

### Sample size determination

The sample size calculation for each group was 36.8 with level of significance of 0.05, a power of 0.9, an effect size of 0.8 and standard deviation of 1.2 [15]. One hundred seventy-eight subjects were included in the study after careful screening of patients reporting to Department of Conservative Dentistry and Endodontics, Universal College of Medical Sciences, Bhairahawa, Nepal.

The preoperative direct digital (Radiovisiography, Gendex Corporation, Cusano Milanino, Milano, Italy) intraoral radiographic examination and clinical records were collected from all the patients, like preoperative pain, pulpal status, periapical status and group of the teeth (anterior or posterior teeth of any of the arch). The pulpal status was checked with electric pulp tester (Digitest™ Parkell Inc., USA) and Endofrost (Coltene/Whaledent GmbH and Co.KG). This was later reconfirmed upon access opening i.e. presence of bleeding and on sensibility tests positive response implied for vital tooth similarly for non-vital tooth absence of bleeding and negative response on sensibility tests. The periapical status was checked by percussion, palpation and bite test. The cases diagnosed as symptomatic/asymptomatic irreversible pulpitis, pulp necrosis without periapical radiolucency and symptomatic apical periodontitis without periapical radiolucency were included in this study. Whereas symptomatic apical periodontitis with periapical radiolucency, asymptomatic apical periodontitis (which always shows periapical radiolucency), acute apical abscess and chronic apical abscess were excluded. The preoperative pain scores for eligible cases were recorded and noted in Numeric pain rating scale (NRS-11).

The 0–10 Numerical Rating Scale (NRS-11) was used in which patients were asked to mark the number between 0 and 10 that matched best to their pain intensity. Zero represents ‘no pain at all’, 1–3 mild pain, 4–6 moderate and 7–10 severe pain. Preoperative pain scores were rated in the clinic and postoperative pain scores at 1st, 2nd and 7th day were recorded by patients at home. Patients were instructed to record pain scores before analgesic intake if required. The response proforma was collected on 7th day of follow-up.

**Fig. 1:**
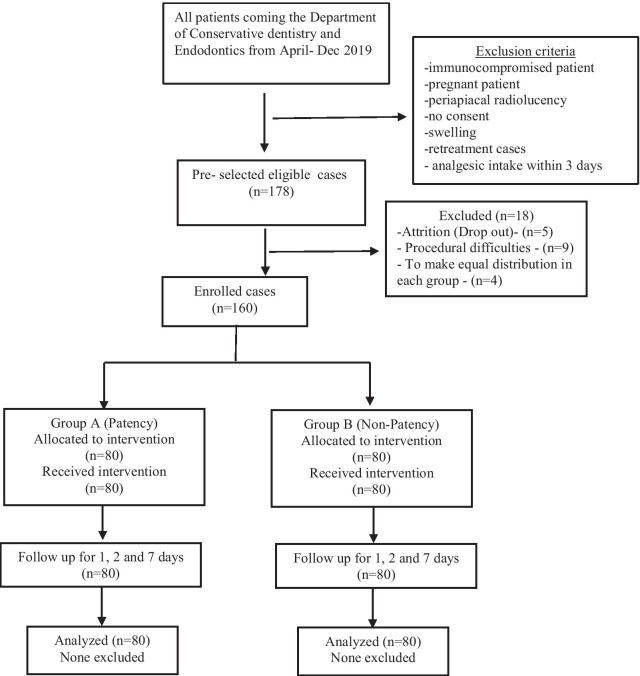
Flowchart showing patients selection and progress

The patients visiting the Department of Conservative Dentistry and Endodontics were assessed for inclusion and exclusion criteria. Among those, 178 patients were preselected with almost equal subjects on the basis of group of teeth (anterior and posterior) and pulp status (vital and non-vital) and were enrolled in the study as shown in Fig. 1. These subjects were randomly allocated to one of the two groups: patency (Group A) and non-patency (Group B). An equal proportion of randomization allocation ratio for the two groups was done by shuffled deck of cards with number assigned (i.e. even number for patency group and odd number for non-patency group). Number of visits were allocated by another set of equal proportion of envelopes containing concealed assignment codes. After the start of the procedure, 18 cases were excluded again due to drop out (n = 5), procedural difficulties (n = 9) and to make equal cases in subgroups (n = 4). The final sample was 160 patients. Endodontic treatments were performed by one endodontist in single and multiple visits and another blinded investigator evaluated and compiled the data.

### Group stratification

Group A: Patency group (n = 80).

- Sub-group A1: Single Visit (n = 40).
  - Sub-division A1V: Vital teeth (n = 20).
    - Division A1Va: Anterior teeth (n = 10).
    - Division A1Vp: Posterior teeth (n = 10).
  - Sub-division A1NV: Non-vital teeth (n = 20).
    - Division A1NVa: Anterior teeth (n = 10).
    - Division A1NVp: Posterior teeth (n = 10).
- Sub-group A2: Multiple Visits (n = 40).
  - Sub-division A2V: Vital teeth (n = 20).
    - Division A2Va: Anterior teeth (n = 10).
    - Division A2Vp: Posterior teeth (n = 10).
  - Sub-division A2NV: Non-vital teeth (n = 20).
    - Division A2NVa: Anterior teeth (n = 10).
    - Division A2NVp: Posterior teeth (n = 10).

Group B: Non-patency group (n = 80).

- Sub-group B1: Single Visit (n = 40).
  - Sub-division B1V: Vital teeth (n = 20).
    - Division B1Va: Anterior teeth (n = 10).
    - Division B1Vp: Posterior teeth (n = 10).
  - Sub-division B1NV: Non-vital teeth (n = 20).
    - Division B1NVa: Anterior teeth (n = 10).
    - Division B1NVp: Posterior teeth (n = 10).
- Sub-group B2: Multiple Visits(n = 40).
  - Sub-division B2V: Vital teeth (n = 20).
    - Division B2Va: Anterior teeth (n = 10).
    - Division B2Vp: Posterior teeth (n = 10).
  - Sub-division B2NV: Non-vital teeth (n = 20).
    - Division B2NVa: Anterior teeth (n = 10).
    - Division B2NVp: Posterior teeth (n = 10).

Local anesthesia (2% lidocaine hydrochloride and epinephrine 1:200,000; Neon laboratories Ltd., India) using conventional nerve block techniques was given to all patients for ease of discomfort. Root canal procedure was started as per standard protocol under rubber dam isolation for all teeth. The access cavity preparation was done using round bur (SS White, USA) and Endo Z bur (Dentsply Maillefer, Switzerland) with highspeed handpiece. Number 8 and 10 K file (Dentsply Maillefer, Switzerland) was used to negotiate the canals with the help of Glyde (Dentsply Maillefer, Switzerland). The Working length (WL) was determined using Propex II apex locator (Dentsply Maillefer, Switzerland) and then confirmed radiographically with 15 K file. If there was disagreement between radiographic and electronic working length measurements, reading of the apex locator was selected.

The root canal treatment was done in either single or multiple visits. Cleaning and shaping of canals were done with hand K-files (Dentsply Maillefer, Switzerland) using step-back technique. For patency group (i.e. Group A), a size 10 K-file was passed 1 mm beyond the working length. The patency technique was done after each file used for step-back technique (i.e. sequential apical preparation files as well as sequential coronal preparation files). Likewise, for non-apical patency group (i.e. Group B) filing was done cautiously to prevent surpassing of patency file beyond the working length at all times during treatment. For both patency and non-patency groups, each root canal was syringe irrigated with 31-gauge, double side vented needle (NaviTip, Ultradent) located 3 mm short of the WL with 5 ml of 5% sodium hypochlorite (NaOCl) solution (Dentpro, India) followed by sterile 0.9% saline (Axa Parenterals Ltd, India), and 2% chlorhexidine (Dentochlore, Ammdent, India) after each instrument change. Throughout the irrigation, needle binding to canal was prevented and the rate was kept constant at 0.25 ml/s to avoid extrusion into periapex (21).

For multiple visit (Group A2 and B2) cases, canal was medicated with calcium hydroxide (RC CAL Prime Dental products ltd, India), temporarily sealed with cavit (3 M ESPE) and recalled after a week for follow-up. In single visit (Group A1 and B1) and asymptomatic multiple visit (Group A2 and B2) cases, the obturation was done in following steps: The master cone radiograph was taken to re-confirm the length. Canals were dried using paper points. AH Plus sealer (Dentsply Maillefer, Ballaigues, Switzerland) was applied on the walls of the canal. After that, obturation was done with lateral condensation technique and access cavity was restored using direct composite resin. Postoperative pain scores were recorded on Numeric pain rating scale forms by the patients on 1st, 2nd and 7th day.

Normality of the data was tested using Kolmogorov Smironov test. The data were not distributed normally hence, non-parametric test i.e. Mann–Whitney U test was used. The relation between pre-operative pain and post-operative pain was evaluated by Spearman correlation test. Multiple linear regression analysis was done to assess the predictor of postoperative pain in patency-maintained group.

## Results

### **Primary outcome**

Statistically significant difference of pain scores was present in Group A (patency group) and Group B (non -patency group) with higher mean rank of pain scores in group A than group B in 1st, 2nd and 7th day (Table 1).

**Table 1:** Comparison of Mean ± SD and Mean Rank of pain score between Patency (Group A) and Non-patency group (Group B)

Follow-up	Group (n = 80)		Group B (n = 80)		*p* value*
	Mean Rank		Mean Rank		
Day 1	88.23		72.77		0.033
Day 2	87.98		73.03		0.036
Day 7	86.68		74.32		0.031

*Mann Whitney U test, Significant at the 0.05 level

### Secondary outcome

When number of visits was considered for postoperative pain in both groups (A and B), no statistically significant difference (*p* > 0.05) in mean rank of pain scores was observed between subgroups: single visit (A1 vs B1) and multiple visits (A2 vs B2) during follow up on 1st, 2nd and 7th day. However, the mean rank of pain scores was higher for patency groups (Group A1, A2) than non-patency groups (Group B1, B2) in all three days (Table 2).

**Table 2:** Comparison of Mean ± SD and Mean Rank of pain score between Patency (Group A) and non-patency group (Group B) for single visit and multi visit

Visits	Variables	Mean Rank		Mann–Whitney U	*p* value*
		Group A (n = 40)	Group B (n = 40)		
Single (1) (n = 80)	Day 1	43.96	37.04	661.500	0.179
	Day 2	45.39	35.61	604.500	0.050
	Day 7	43.45	37.55	682.000	0.140
Multiple (2) (n = 80)	Day 1	44.89	36.11	624.500	0.089
	Day 2	43.29	37.71	688.500	0.274
	Day 7	43.75	37.25	670.000	0.116

*Mann Whitney U test, Significant at the 0.05 level

When vital and non-vital teeth were treated in single visit, no statistically significant difference of mean rank of pain scores was observed between patency maintained (A1V, A1NV) to not maintained group (B1V, B1NV) with higher mean rank of pain scores for patency group (A1V, A1NV) in 1st, 2nd and 7th day (Table 3).

**Table 3:** Comparison of Mean ± SD and Mean Rank of pain score between Patency (Group A) and non-patency group (Group B) for single visit and multi visit vital and non-vital tooth respectively

Visits/Status	Variables	Mean Rank		Mann–Whitney U	*p* value*
		Group A (n = 20)	Group B (n = 20)		
Single/vital (1 V) (n = 40)	Day 1	21.00	20.00	190.000	0.799
	Day 2	21.83	19.18	173.500	0.478
	Day 7	21.65	19.35	177.000	0.547
Multiple/vital (2 V) (n = 40)	Day 1	24.78	16.23	114.500	0.020
	Day 2	23.70	17.30	136.000	0.086
	Day 7	23.25	17.75	145.000	0.142
Single/non-vital (1NV) (n = 40)	Day 1	23.40	17.60	142.000	0.121
	Day 2	24.05	16.95	129.000	0.056
	Day 7	22.35	18.65	163.000	0.327
Multiple/non-vital (2NV) (n = 40)	Day 1	20.05	20.95	191.000	0.820
	Day 2	19.78	21.23	185.500	0.698
	Day 7	20.90	20.10	192.000	0.841

*Mann Whitney U test, Significant at the 0.05 level

When vital and non-vital teeth were treated in multiple visits, mean rank of pain scores were higher for vital teeth of patency group (A2V) than non-patency group (B2V) in 1st, 2nd and 7th days with statistically significant difference (*p* < 0.05) in day 1. Whereas, for non-vital teeth even though the mean rank of pain scores were lower for patency group (A2NV) in day 1 and 2, the scores were statistically non-significant (Table 3).

When patency was maintained, the post-operative pain scores of anterior teeth (A1Va, A1NVa, A2Va, A2NVa) versus posterior teeth (A1Vp, A1NVp, A2Vp, A2Vp) was statistically non-significant (Table 4). The result was similar for non-patency group of teeth (Table 5).

**Table 4:** Comparison of Mean ± SD and Mean Rank of pain score between anterior and posterior tooth for single visit and multi visit vital and non-vital tooth respectively when apical patency was maintained

Visits/Status	Variables	Mean Rank (n = 10)		Mann–Whitney U	*p* value*
		Anterior (a) (n = 10)	Posterior (p) (n = 10)		
Single/vital (A1V) (n = 20)	Day 1	10.40	10.60	49.000	0.971
	Day 2	9.85	11.15	43.500	0.631
	Day 7	10.50	10.50	50.000	1.000
Single/non-vital (A1NV) (n = 20)	Day 1	11.50	9.50	40.000	0.481
	Day 2	9.75	11.25	42.500	0.579
	Day 7	9.50	11.50	40.000	0.481
Multiple/vital (A2V) (n = 20)	Day 1	9.55	11.45	40.500	0.467
	Day 2	8.95	12.05	34.500	0.234
	Day 7	9.20	11.80	37.000	0.295
Multiple/non-vital (A2NV) (n = 20)	Day 1	9.50	11.50	40.000	0.481
	Day 2	8.80	12.20	33.000	0.218
	Day 7	9.50	11.50	40.000	0.481

*Mann Whitney U test, Significant at the 0.05 level

**Table 5:** Comparison of Mean ± SD and Mean Rank of pain score between anterior and posterior tooth for single visit and multi visit vital and non-vital tooth respectively when apical patency was not maintained

Visits/status	Variables	Mean Rank		Mann–Whitney U	*p* value*
		Anterior (a) (n = 10)	Posterior (p) (n = 10)		
Single-vital (B1V) (n = 20)	Day 1	9.70	11.30	42.000	0.579
	Day 2	9.75	11.25	42.500	0.579
	Day 7	9.95	11.05	44.500	0.684
Single/non-vital (B1NV) (n = 20)	Day 1	8.00	13.00	25.000	0.063
	Day 2	10.40	10.60	49.000	0.971
	Day 7	9.50	11.50	40.000	0.481
Multiple/vital (B2V) (n = 20)	Day 1	9.70	11.30	42.000	0.579
	Day 2	10.15	10.85	46.500	0.796
	Day 7	9.70	11.30	42.000	0.579
Multiple/nonvital (B2NV) (n = 20)	Day 1	11.40	9.60	41.000	0.491
	Day 2	10.00	11.00	45.000	0.698
	Day 7	11.05	9.95	44.500	0.503

*Mann Whitney U test, Significant at the 0.05 level

The result of Spearman correlation test showed that the preoperative pain was significantly correlated with post-operative pain in both patency group and non-patency group. There was statistically significant low degree positive correlation between pre-operative pain and post-operative pain in day 1 in both patency group (ρ = 0.285, *p* = 0.01) as well as non-patency group (ρ = 0.576, *p* < 0.001). Whereas, there was statistically significant high degree positive correlation between pre-operative pain and post-operative pain in day 2 in both patency group (ρ = 0.871, *p* < 0.001) as well as non -patency group (ρ = 0.798, *p* < 0.001) (Table 6).

**Table 6:** Correlation of Preoperative pain and Postoperative pain when patency is maintained (Group A) and not maintained (Group B)

Variables	Group A		Group B	
	Correlation (ρ)	*p* value*	Correlation (ρ)	*p* value*
P_0_–P_1_	0.285	0.011	0.576	0.0001
P_1_–P_2_	0.871	0.0001	0.798	0.0001
P_2_–P_7_	0.574	0.0001	0.622	0.0001

P_0_ = Preoperative pain P_1_ = Day 1 pain score P_2_ = Day 2 pain score and P_7_ = Day 7 pain score

*Spearman correlation

When postoperative pain for apical patency was predicted with multiple regression analysis, it was found that status of pulp (Beta= − 0.361, *p* = 0.001), (Beta = − 0.368, *p* = 0.001) and (Beta = − 0.344, *p* = 0.003) at day 1, 2 and 7 respectively were significant predictor. Also, preoperative pain (Beta = 0.346, *p* = 0.002), (Beta = 0.224, *p* = 0.044) at day 1 and 2 respectively were significant predictors (Table 7). The overall model fit was R^2^ = 0.199, 0.191 and 0.134 for day 1, 2 and 7 respectively.

**Table 7:** Linear regression analysis for post-operative pain

Variables	B	Standard error	Beta	*p* value
*Tooth*				
Day 1	0.125	0.570	0.023	0.827
Day 2	0.887	0.501	0.185	0.081
Day 7	0.154	0.167	0.099	0.361
*Status*				
Day 1	− 1.981	0.593	− 0.361	0.001
Day 2	− 1.764	0.521	− 0.368	0.001
Day 7	− 3	0.174	− 0.344	0.003
*Visit*				
Day 1	0.797	0.567	0.145	0.164
Day 2	0.513	0.498	0.107	0.306
Day 7	0.177	0.166	0.114	0.290
*Preoperative pain*				
Day 1	0.445	0.140	0.346	0.002
Day 2	0.251	0.123	0.224	0.044
Day 7	0.047	0.041	0.128	0.259

## Discussion

The present study aimed to assess postoperative pain while maintaining apical patency. Pain is a subjective sign, so it is difficult to assess accurately and quantify in any statistical analysis [22] so it is crucial to select proper pain assessment tool. VAS (Visual Analogue Scale) was used in many studies [16–18] as pain assessment tool for postoperative pain when patency was maintained whereas NRS-11 scale was used in our study as it has high reliability and validity [23]. A systematic review in 2011, authors found that even though the studies were inconclusive regarding preference for a particular tool, the NRS was considered superior in 11 studies and the VAS was recommended in only four studies [24].

Bi-dimensional radiographic technique was used for preoperative assessment of root canal and periapical status. But it doesn’t always accurately reflect normal and periapical pathology. Periapical inflammatory lesions can go unnoticed especially in the early stages, even in the late stages, and depending on the type of bone, such as cancellous or cortical [25]. Hence, to overcome potential shortcomings, three-dimensional radiograph is preferred choice but high radiation exposure, lengthy scan time and cost factor limits its use [26]. Determination of working length accurately was also essential for the present study. It was determined with Propex II electronic apex locator as well as with a radiograph. Propex II apex locator was used as its accuracy is comparable to Root ZX apex locater [27, 28].

The file size #10 K was used to maintain apical patency in this study to ensure least apical enlargement and transportation, decreased extrusion of debris and less injury of periapical tissues as all these effects increase the incidence of post-endodontic pain and risk the outcome of treatment [29, 30]. Post-operative pain is also affected by irrigation mechanics hence manual syringe irrigation with small gauge, double side vented needle placed at 3 mm from WL was used to ensure apical cleaning as well as decrease periapical extrusion [21].

In our study, mean rank of postoperative pain scores was higher in apical patency group than non-patency group in 1st, 2nd and 7th day follow up with statistically significant difference. The result is in contrast to other studies [14–20]. Arias et al. [14], Garg et al. [16] and Sharaan et al. [17] reported maintaining apical patency did not increase post-operative pain whereas Arora et al. [15] and Yaylali et al. [18] found decrease in post-operative pain when patency was maintained. Even systematic review [19] and meta-analysis [20] also concluded that maintaining apical patency did not increase postoperative pain.

The contradictory result of patency group with increase in post-operative pain might be due to disruption of apical constriction leading to increase extrusion of debris of the canal into periapical tissue [29, 31]. Torabinejad et al. [32] also stated intentional over-instrumentation of files may disrupt the apical stop leading to extrusion of filling materials. The greater periapical extrusion of debris might be enhanced by manual filing technique as supported by Deonizio et al. [33]. In addition to this, the variation of results might be due to difference in filing technique, file system and difference in pain perception by different population. In most of the studies shaping and cleaning was done with rotary instruments [15–18] whereas shaping and cleaning in our study was done by manual K files with step back technique which requires more frequent number of filing and recapitulations, increasing the possibility of greater extrusion and peri-apical injury thereby increasing the chance for more post-operative pain in an attempt to maintain apical patency.

Our secondary outcome was to evaluate the influence of number of visits, status of pulp, type of teeth and preoperative pain on post-operative pain as literature has shown these factors intensify inflammatory reaction leading to pain [31, 34, 35].

The studies [14, 16] in which apical patency was maintained in single visit RCT, showed no significant difference in post-operative pain scores with non-patency group. Whereas, Yaylali et al. [18] reported higher mean pain scores for non-patency group compared to patency group. In addition, the studies in which apical patency was maintained in multiple visit RCT, result showed variable results such as increase in post-operative pain in 6 and 12 h follow up in patency group compared to non-patency group [17] whereas, study done by Arora et al. [15] showed less pain in apical patency group in 1st, 2nd, 3rd and 4th day follow up. These studies have evaluated postoperative pain in either single or multiple visits RCT, whereas literature search has revealed paucity of research comparing postoperative pain after maintaining apical patency in both single visit and multiple visit. Our study compared post-operative pain in single visit RCT and multiple visit RCT after patency maintenance and non-maintenance, the result showed no significant difference in mean rank of pain scores in follow up of day 1, 2 and 7 as well as regression analysis also showed visits as non-significant predictor.

When pulpal status was concerned, postoperative pain scores were statistically non-significant in patency group versus non-patency group in single visit treatment in day 1, 2 and 7 with higher mean rank of pain scores in non-vital teeth. The result was coherent to the study done by Siqueira et al. [31] which showed non-vital teeth more prone for post-operative pain than vital teeth due to extrusion of infected debris in addition to the direct mechanical trauma caused by instruments leading to more severe periapical inflammatory response [36].

But, in multiple visit treatment, the mean rank of pain scores was higher for vital teeth of patency group than non-patency group in 1st, 2nd and 7th days with statistically significant difference (*p* < 0.05) in day 1 similar to the study done by Arias et al. [14]. The higher score in vital teeth might be due coronal leakage in multiple visit pertaining prolonged inflammatory response with highest inflammatory response within 24 h of periapical injury.

The result of present study showed that postoperative pain is not influenced by the type of tooth (i.e. anterior or posterior) treated by apical patency maintenance or non-maintenance which can be explained by the fact that postoperative pain is dependent upon the amount of periapical injury followed by inflammatory response and not just with the number of roots. The result is coherent to the study conducted by Arias et al. [14] and Garg et al. [16]. However, studies have found that tooth type did affect the post-operative pain due to complexity of canal morphology and number of canals [32, 34].

When pre-operative pain scores were correlated for postoperative pain, the result showed statistically significant postoperative pain scores in patency group and non-patency groups which is similar to study done by Garg et al. [16] and Elmubarak et al. [37]. This might be due to severe inflammatory response in already inflamed tooth or due to perception of pain in previously sensitized tooth. Whereas, the results were conflicting to studies done by Ng et al. [34] and Albashaireh el al. [38] which showed no effect of pre-operative pain in post-operative pain.

To better predict the influencing factor for postoperative pain in apical patency group, regression analysis was done. Among the four different independent diagnostic factors, status of pulp and pre-operative pain were only significant predictors for postoperative pain. Similarly, status of pulp has an influence on postoperative pain as stated by different studies [38, 39]. Flare-up and postoperative pain are dependent upon the status of the pulp. Due to interaction of microorganisms and peri-apical tissue, non-vital teeth are more prone for it than vital teeth. In addition to microbial injury, chemical and mechanical insults may cumulatively affect for postoperative pain [40]. But, this result is in contrast to study done by Sevekar et al. [41] which showed pretreatment status doesn’t predict the postoperative pain. Another predictor for postoperative pain was preoperative pain, which was similar to other studies [35, 42].

Hence, it can be implied that apical patency does have an effect on postoperative pain. It cannot be imposed upon for apical cleaning as there are several other methods like ultrasonic agitation, negative pressure irrigation, lasers etc. So, rather doing benefit to the patient we are unnecessarily increasing the discomfort to the patient. But to have more conclusive results, study can be done on larger population with comparison of different file systems and longer follow up period.

## Conclusions

The maintenance of apical patency increases postoperative pain. Evaluation of influence of number of visits, status of pulp, group of tooth and preoperative pain revealed pulpal status and preoperative pain as influencing factors for postoperative pain in patency group.

## Data Availability

The datasets used and analyzed during the study are available from the corresponding author on reasonable request.

## Abbreviations

RCT: Root canal treatment
NaOCl: Sodium hypochlorite
PO: Preoperative
NRS: Numeric rating scale
